# GTP-Dependent FlhF Homodimer Supports Secretion of a Hemolysin in *Bacillus cereus*

**DOI:** 10.3389/fmicb.2020.00879

**Published:** 2020-05-06

**Authors:** Diletta Mazzantini, Rossella Fonnesu, Francesco Celandroni, Marco Calvigioni, Alessandra Vecchione, Devid Mrusek, Gert Bange, Emilia Ghelardi

**Affiliations:** ^1^Department of Translational Research and New Technologies in Medicine and Surgery, University of Pisa, Pisa, Italy; ^2^Center for Synthetic Microbiology (SYNMIKRO) and Department of Chemistry, Philipps University, Marburg, Germany

**Keywords:** *Bacillus cereus*, flhF, L2, hemolysin BL, virulence, flagellin

## Abstract

The multidomain (B-NG) protein FlhF, a flagellar biogenesis regulator in several bacteria, is the third paralog of the signal recognition particle (SRP)-GTPases Ffh and FtsY, which are known to drive protein-delivery to the plasma membrane. Previously, we showed that FlhF is required for *Bacillus cereus* pathogenicity in an insect model of infection, being essential for physiological peritrichous flagellation, for motility, and for the secretion of virulence proteins. Among these proteins, we found that the L_2_ component of hemolysin BL, one of the most powerful toxins *B. cereus* produces, was drastically reduced by the FlhF depletion. Herein, we demonstrate that *B. cereus* FlhF forms GTP-dependent homodimers *in vivo* since the replacement of residues critical for their GTP-dependent homodimerization alters this ability. The protein directly or indirectly controls flagellation by affecting flagellin-gene transcription and its overproduction leads to a hyperflagellated phenotype. On the other hand, FlhF does not affect the expression of the L_2_-encoding gene (*hblC*), but physically binds L_2_ when in its homodimeric form, recruiting the protein to the plasma membrane for secretion. We additionally show that FlhF overproduction increases L_2_ secretion and that the FlhF/L_2_ interaction requires the NG domain of FlhF. Our findings demonstrate the peculiar behavior of *B. cereus* FlhF, which is required for the correct flagellar pattern and acts as SRP-GTPase in the secretion of a bacterial toxin subunit.

## Introduction

*Bacillus cereus* is a Gram-positive, motile, and spore-forming rod that is ubiquitously found in a wide range of environments including soil, water and foods. Long known as cause of two types of food-borne intoxications, the diarrheal and emetic syndromes, *B. cereus* is also responsible for local and systemic infections in humans (Ehling-Schulz et al., 2019). Its pathogenicity is associated with the bacterial ability to adhere to and colonize host surfaces through peritrichous flagella and flagellum-driven swimming and swarming (Senesi and Ghelardi, 2010). Beside motility, the secretion of tissue-destructive exoenzymes and toxins, e.g. hemolysins, phospholipases, trimeric toxins, cytotoxins and proteases (Ramarao and Sanchis, 2013; Ehling-Schulz et al., 2019), is of crucial importance in *B. cereus* pathogenicity. Among these factors, the tripartite hemolysin BL (HBL), consisting of a binding component (B) and two lytic components (L_1_ and L_2_), plays a primary role in diarrheal food poisoning and necrotizing infections such as endophthalmitis, due to its vascular permeability, hemolytic, and enterotoxic activity (Beecher et al., 1995a, b). Flagella, swarming differentiation, and HBL secretion are intrinsically linked in *B. cereus*. In fact, HBL export is increased in hyperflagellated swarm cells (Ghelardi et al., 2007; Salvetti et al., 2011) and mutations that reduce the number of flagella impact on toxin secretion (Senesi et al., 2002; Callegan et al., 2006; Salvetti et al., 2007; Mazzantini et al., 2016). In addition, failure to secrete HBL was demonstrated in aflagellated *B. cereus* natural isolates (Ghelardi et al., 2007) and in an acrystalliferous *Bacillus thuringiensis* (i.e. *B. cereus*) mutant for the Type-III flagellar export apparatus (Ghelardi et al., 2002).

The third signal recognition particle (SRP) GTPase, FlhF, is involved in controlling *B. cereus* flagellation and motility (Salvetti et al., 2007; Mazzantini et al., 2016). In many polarly flagellated species, this protein is required for establishing the correct place and/or quantity of flagella on the cell surface (Kazmierczak and Hendrixson, 2013; Altegoer et al., 2014; Gao et al., 2015; Schuhmacher et al., 2015; Navarrete et al., 2019), together with the MinD-like ATPase FlhG (Leipe et al., 2002). In the peritrichously flagellate *Bacillus subtilis*, FlhF and FlhG behave as antagonistic pair in regulating the symmetric grid-like pattern of flagellar basal body distribution, but do not control the number of flagella on the cell surface (Bange et al., 2011; Guttenplan et al., 2013). In *B. cereus*, no FlhG homolog was found and depletion of FlhF causes flagellar mislocalization and substantial reduction in the number of flagella (Salvetti et al., 2007).

Together with Ffh and FtsY, FlhF belongs to the SRP family of GTPases (Leipe et al., 2002). In the canonical SRP system, the Ffh/4.5S RNA complex attaches to the N-terminal signal sequence of nascent membrane protein at the ribosome, and then docks with its receptor (FtsY) on the membrane, thus resulting in the insertion of the protein into the membrane (Akopian et al., 2013; Mercier et al., 2017). SRP-GTPases are characterized by conserved N and G domains that form a structurally and functionally coupled unit (NG domain) (Grudnik et al., 2009). In all described SRP-GTPases, five nucleotide-binding signature elements (G1–G5) essential for GTP binding and hydrolysis and for SRPs dimerization have been identified in the G domain (Bange et al., 2007; Mazzantini et al., 2016). All three SRP-GTPases contain additional domains, which reflect the specific functional roles of these proteins. While Ffh presents a C-terminal methionine-rich domain (M) that is essential for signal peptide recognition and SRP-RNA binding (Janda et al., 2010; Hainzl et al., 2011), FtsY contains an unfolded N-terminal acidic domain (A) which ensures membrane attachment (de Leeuw et al., 2000; Stjepanovic et al., 2011; Altegoer et al., 2014). Similar to *B. subtilis* FlhF (Bange et al., 2007), the *B. cereus* protein carries a natively unfolded N-terminal lysine-rich basic (B) domain with unknown function (Mazzantini et al., 2016). GTP-bound FlhF forms homodimers in *Xantomonas oryzae* pv *oryzae*, *B. subtilis*, *Pseudomonas aeruginosa*, *Shewanella oneidensis*, *Campylobacter jejuni*, and *Vibrio alginolyticus* (Shen et al., 2001; Bange et al., 2007; Schniederberend et al., 2013; Gao et al., 2015; Gulbronson et al., 2016; Kondo et al., 2018).

In addition to the flagellar defects, FlhF depletion in *B. cereus* also causes an alteration in the amount of several extracellular proteins (Mazzantini et al., 2016). Among these proteins, the L_2_ component of HBL was found to be consistently reduced in the extracellular proteome of a *B. cereus* Δ*flhF* mutant (Mazzantini et al., 2016), suggesting a potential involvement of FlhF in L_2_ secretion mechanisms.

In the present study, the ability of *B. cereus* FlhF to form homodimers and the effect of point mutations in FlhF putative GTP-binding elements on homodimer formation were analyzed. Functional characterization of *B. cereus* FlhF as SRP protein for L_2_ is reported and the requirement of the NG domain for protein contact is defined. In addition, experiments were performed to investigate the effect of different FlhF levels on *B. cereus* flagellation and L_2_ secretion.

## Materials and Methods

### Bacterial Strains and Culture Conditions

All strains used in this study are described in Table 1. *B. cereus* ATCC 14579 wild-type (WT), its *flhF* mutant (Δ*flhF*, MP06), and *flhF*-overexpressed (MP08) derivatives (Salvetti et al., 2007) were grown at 37°C in Brain Heart Infusion (BHI) supplemented with 1% glucose (BHIG). When required, 5 μg/ml erythromycin for strain Δ*flhF* or 30 μg/ml kanamycin for strain MP08 selection were added. MP08 cultures were also supplemented with 4 mM isopropyl-β-D-1-tiogalattopiranoside (IPTG; Merck KGaA, Darmstadt, Germany) to induce *pspac*-dependent *flhF* expression. *E. coli* XL1-Blue was used for general cloning strategies, *E. coli* BTH101 was used as reporter strain in the bacterial adenylate cyclase two-hybrid system (BACTH) assays, and *E. coli* BL21 (DE3) for hexa-histidine pull-down experiments. *E. coli* strains were grown at 30°C or 37°C in Luria Bertani (LB) supplemented with 100 μg/ml of ampicillin, 50 μg/ml of kanamycin, or either, when required. *E. coli* BTH101 and BL21 (DE3) cultures were also supplemented with 0.5 and 1 mM IPTG, respectively, to induce plasmid gene expression.

**TABLE 1:** Strains and plasmids used in this study.

Strain	Relevant genotype or description	Source or references
***B. cereus***		
ATCC 14579 (WT)	Wild-type strain	ATCC
Δ*flhF* (MP06)	Derivative of ATCC 14579, carrying a Campbell integration of pRND*flhF*2 in *flhF*	Salvetti et al., 2007
MP08	Derivative of ATCC 14579, containing pDG*flhF*	Salvetti et al., 2007
***E. coli***		
XL1-Blue	endA1 gyrA96(nal^R^) thi-1 recA1 relA1 lac glnV44 F’[ ::Tn10 proAB^+^ lacI^q^ Δ(lacZ)M15] hsdR17(r_K_^–^ m_K_^+^); used for subcloning	Stratagene, La Jolla, California
BTH101	F-, cya-99, araD139, galE15, galK16, rpsL1 (Str^R^), hsdR2, mcrA1, mcrB1	Euromedex, Souffelweyersheim, France
BL21 (DE3)	B F– ompT gal dcm lon hsdSB(rB^–^mB^–^) λ(DE3 [lacI lacUV5-T7p07 ind1 sam7 nin5]) [malB^+^]_K–12_(λ^S^)	Thermo Fisher Scientific

**Plasmids**	**Description**	**Source**

**For BACTH experiments**		
pUT18	Derivative of pUC19, ori *E. coli* ColE1, Amp^R^, *plac*-MCS (HindIII–SphI–PstI–XbaI–BamHI–SmaI–KpnI–SacI–EcoRI)-T18	Euromedex
pUT18-*flhF*	Derivative of pUT18 expressing FlhF-T18 fusion	This study
pUT18-*flhF*_NG_	Derivative of pUT18 expressing FlhF_NG_-T18 fusion	This study
pUT18-*bc1657*	Derivative of pUT18 expressing BC1657-T18 fusion	This study
pUT18-*hblC*	Derivative of pUT18 expressing L_2_-T18 fusion	This study
pUT18-*flhF*_T253Q_	Derivative of pUT18 expressing FlhF_T253Q_-T18 fusion	This study
pUT18-*flhF*_D391A_	Derivative of pUT18 expressing FlhF_D391A_-T18 fusion	This study
pUT18C	Derivative of pUC19, ori *E. coli* ColE1, Amp^R^, *plac*-T18-MCS (HindIII–SphI–PstI–XbaI–BamHI–SmaI–KpnI–SacI–EcoRI)	Euromedex
pUT18C-*zip*	pUT18C expressing the T18-leucine zipper motif of GCN4 fusion	Euromedex
pUT18C-*flhF*	Derivative of pUT18C expressing T18-FlhF fusion	This study
pUT18C-*flhF*_NG_	Derivative of pUT18C expressing T18-FlhF_NG_ fusion	This study
pUT18C-*bc1657*	Derivative of pUT18 expressing T18-BC1657 fusion	This study
pUT18C-*hblC*	Derivative of pUT18 expressing T18-L_2_ fusion	This study
pUT18C-*flhF*_T253Q_	Derivative of pUT18 expressing T18-FlhF_T253Q_ fusion	This study
pUT18C-*flhF*_D391A_	Derivative of pUT18 expressing T18-FlhF_D391A_ fusion	This study
pKT25	Derivative of pSU40, ori *E. coli* p15A, Km^R^, *plac*-T25-MCS (PstI–XbaI–BamHI–SmaI–KpnI–EcoRI)	Euromedex

**Strain**	**Relevant genotype or description**	**Source or references**

pKT25-*zip*	Derivative of pKT25 expressing T25-leucine zipper motif of GCN4 fusion	Euromedex
pKT25-*flhF*	Derivative of pKT25 expressing T25-FlhF fusion	This study
pKT25-*flhF*_NG_	Derivative of pKT25 expressing T25-FlhF_NG_ fusion	This study
pKT25-*bc1657*	Derivative of pKT25 expressing T25-BC1657 fusion	
pKT25-*hblC*	Derivative of pKT25 expressing T25-L_2_ fusion	This study
pKT25-*flhF*_T253Q_	Derivative of pKT25 expressing T25-FlhF_T253Q_	This study
pKT25-*flhF*_D391A_	Derivative of pKT25 expressing T25-FlhF_D391A_ fusion	This study
pKNT25	Derivative of pSU40, ori *E. coli* p15A, Km^R^, *plac* -MCS (PstI–XbaI–BamHI–SmaI–KpnI–EcoRI)-T25	Euromedex
pKNT25-*flhF*	pKNT25 expressing leucine zipper motif of GCN4-T25 fusion	This study
pKNT25-*flhF*_NG_	pKNT25 expressing FlhF_NG_-T25 fusion	This study
pKNT25-*bc1657*	pKNT25 expressing BC1657-T25 fusion	This study
pKNT25-*hblC*	pKNT25 expressing L_2_-T25 fusion	This study
pKNT25-*flhF*_T253Q_	pKNT25 expressing FlhF_T253Q_-T25 fusion	This study
pKTN25-*flhF*_D391A_	pKNT25 expressing FlhF_D391A_-T25 fusion	This study
**For hexa histidine pull-down experiments**		
pET303/CT-His	ori *E. coli* pBR322 derived, Amp^R^, T7*lac*-RBS-MCS (XbaI-NsiI-XhoI)-6 × His Tag-T7 terminator	Thermo Fisher Scientific
pET303/*flhF*_His_	Derivative of pET303/CT-His expressing FlhF-6 × His tag (FlhF_His_) fusion	This study
pET303/*hblC*	Derivative of pET303/CT-His expressing L_2_	This study

### Plasmids Construction

Plasmids and primers used in this study are described in Table 1 and Supplementary Table S1, respectively. For bacterial two-hybrid experiments, the complete ORFs of *B. cereus flhF* (GenBank ID: *bc1670*), *hblC* (GenBank ID: *bc3104*), *bc1657*, and the NG encoding fragment of *flhF* (*flhF*_NG_) were directly amplified from genomic DNA of *B. cereus* ATCC 14579 using primer pairs *flhF*F1/*flhF*R1, *bc1657*F1/*bc1657*R1, L_2_F2/L_2_R1, NGF_1_/*flhF*R1, respectively. To produce *flhF*_T253Q_ and *flhF*_D391A_ mutant variants, site-directed mutagenesis by combined overlap extension PCR (COE-PCR) was performed (Hussain and Chong, 2016). For each variant, two primers pairs (*BcflhF*-T253Q-F/*flhF*R1 and *flhF*F1/*BcflhF*-T253Q-R, and *BcflhF*-D391A-F/*flhF*R1 and *flhF*F1/*BcflhF*-D391A-R, respectively), were used to generate two *flhF* mutated fragments with overlapping ends. The full-length products were obtained directly joining the two fragments by PCR using *flhF*F1/*flhF*R1 as primer pair. All amplicons were digested with BamHI/KpnI (New England Biolabs, NEB, Ipswich, MA, United States), and ligated in pUT18, pUT18C, pKT25, and pKNT25 vectors, in *frame* with the isolated T18 and T25 domains of adenylate cyclase and under the control of the inducible *plac* promoter. For hexa-histidine pull-down assays, *flhF* and *hblC* were amplified using PD*flhF*F1/PD*flhF*R2 and PDL_2_F1/PDL_2_R1, digested with XbaI/XhoI and NsiI/XhoI (NEB), respectively, and ligated in pET303/CT-His, under the control of the T7 promoter. Recombinant vectors were propagated in *E. coli* XL1-Blue and analyzed by PCR, plasmids extraction, and sequencing.

### Quantitative Real Time PCR

WT and Δ*flhF* cells were inoculated in 100 ml of BHIG broth and grown at 37°C until mid-exponential phase (OD_600_ of ∼1.0). Bacterial cultures were centrifuged at 4,000 × *g* at 4°C for 15 min. Cells were washed twice with cold diethylpyrocarbonate-treated water and suspended in 350 μl of RA1 lysis buffer (Macherey-Nagel, Düren, Germany) supplemented with 3.5 μl of β-mercaptoethanol and 0.35 g of zirconiabeads (diameter 0.1 mm; Biospec Products, Barltesville, Okla). Bacterial lysis was performed by shaking for 4 min with a mini-bead beater (Biospec Products), alternating 0.5 min of shake with 5 min in ice bath. Residual cells and debris were removed by centrifugation for 2 min at 12,000 × *g* and the aqueous phases were filtered through NucleoSpin^®^ Filters. 350 μl of 70% absolute ethanol were added, and the mixture was applied to a NucleoSpin^®^ RNA column. After being digested with 40 units of RNase-free DNase (Macherey-Nagel), total RNA was eluted from the column following the manufacturer’s instructions. 500 ng of the purified RNA were used as a template in one-step RT-PCR with the TransScript^®^ One-Step gDNA Removal and cDNA Synthesis SuperMix (Transgenbiotech, Beijing, China), according to the manufacturers. qRT-PCR were performed on cDNA samples using the LightCycler^TM^ FastStart DNA Master SYBR Green I and analyzed in the LightCycler instrument (Roche, Basel, Switzerland). The *rpoA* (GenBank ID: *bc0158*) and the *bc4306* genes (Reiter et al., 2011; Salvetti et al., 2011) were used as endogenous controls. qRT-PCRs were performed using the primer pairs *bc1657* Fup/*bc1657* Rdw, hblL_2_ up/hblL_2_ dw, rpoAup1/rpoAdw1, and gatB_Yqey up/gatB_Yqey dw (Supplementary Table S1) for *bc1657*, *hblC*, *rpoA*, and *bc4306*, respectively. The amplification conditions were optimized and the amplified fragments sequenced (Supplementary Table S1) before performing qRT-PCR experiments. Melting curve analysis was carried out in parallel to qRT-PCR to confirm the specificities of the amplification reactions. Data were analyzed using the 2^–ΔΔ*CT*^ method (Livak and Schmittgen, 2001). Three biological replicates were performed and for each experiment three technical replicates were carried out.

### Bacterial Adenylate Cyclase Two-Hybrid Assay

Bacterial adenylate cyclase two-hybrid assay experiments were performed according to Battesti and Bouveret (2012). The constructed BACTH vectors (Table 1) were co-transformed in chemical competent *E. coli* BTH101 cells and plates were incubated at 30°C for 48 h. For each transformation, colonies were inoculated in 3 ml of LB supplemented with 100 μg/ml ampicillin, 50 μg/ml kanamycin, and 0.5 mM IPTG, and grown overnight at 30°C. 10 μl of each culture were dropped on M63 synthetic medium (2 g/l (NH_4_)_2_SO_4_, 13.6 g/l KH_2_PO_4_, 0.5 mg/l FeSO_4_ × 7H_2_O, 1 mM MgSO_4_, 0.0001% thiamin, 0.2% maltose; pH 7.0) agar plates supplemented with 50 μg/ml ampicillin, 25 μg/ml kanamycin, 40 μg/ml 5-bromo-4-chloro-3-indolyl-β-d-galactopyranoside (X-Gal; Merck KGaA), and 0.5 mM IPTG. Plates were incubated at 30°C for 24–72 h. At least three biological replicates were performed and a representative result is shown.

### Quantification of β-Galactosidase Activity

β-galactosidase assays were performed according to the Miller’s method (Miller, 1992). Overnight bacterial cultures were diluted in 10 ml of LB broth containing 100 μg/ml ampicillin, 50 μg/ml kanamycin, and 0.5 mM IPTG and grown at 30°C to OD_600_ of ∼0.5. 1 ml of each culture was centrifuged at 8000 × *g* at 4 °C for 5 min and the pellet was suspended in an equal amount of chilled Z buffer (60 mM Na_2_HPO_4_ × 7H_2_O, 40 mM NaH_2_PO_4_ × H_2_O, 10 mM KCl, 1 mM MgSO4 × 7H_2_O, and 50 mM β-mercaptoethanol; pH 7.0). For cells permeabilization, 20 μl of 0.1% sodium dodecyl sulfate (SDS; Merck KGaA) and 40 μl of chloroform were added and the tubes were mixed by vortexing for 10 s. 100 μl of samples were diluted in 1 ml of chilled Z buffer and incubated with 200 μl of 4 mg/ml orto-nitrofenil-β-galactopyranoside (ONPG, Merck KGaA) at 28°C. The reaction was stopped by adding 250 μl of 1 M Na_2_CO_3_. β-galactosidase activity was calculated by the Miller formula (Miller units = 1000 × (OD_420_-(1.75 × OD_550_)/T × V × OD_600_); T, reaction time; V, volume of culture assayed in milliliter). Experiments were repeated three times in separate days and for each assay two technical replicates were carried out.

### Hexa-Histidine Pull-Down Experiments

pET303/CT, pET303/*flhF*_His_ and pET303/*hblC* (Table 1) were independently transformed in chemical competent *E. coli* BL21 (DE3). Bacteria were grown at 37°C in 100 ml of LB broth to OD_600_ of ∼0.5 and genes expression was induced by adding 1 mM IPTG. Cultures were incubated for additional 2 h (OD_600_ of ∼2) and centrifuged at 5,000 × *g* for 5 min at 4°C. Pellets were suspended in 1:1 Tris Buffered Saline (TBS; 50 mM Tris, 150 mM NaCl, pH 7.2) and Pierce Lysis Buffer (Thermo Fisher Scientific, Waltham, MA, United States), and supplemented with Halt Protease Inhibitor Cocktail, EDTA-Free 1 × (Thermo Fisher Scientific). Soluble fractions were isolated by centrifugation at 12,000 × *g* for 5 min and stored at – 20°C until use. The correct production of FlhF_His_ and L_2_ in the respective *E. coli* lysates was assessed by immunoblot using mouse monoclonal Anti-His(C-term)-AP Antibody (a-His; Thermo Fisher Scientific) and rabbit polyclonal sera specific to L_2_ (Beecher et al., 1995b), respectively. The *E. coli* lysate expressing FlhF_His_ was incubated for 1 h at 4°C with 25 ml of settled HisPur^TM^ Cobalt resin (Thermo Fisher Scientific), previously equilibrated according to the manufacturer’s instructions. After six washing steps with 1:1 TBS and Pierce Lysis Buffer containing 20 mM imidazole (Thermo Fisher Scientific), the *E. coli* lysate producing L_2_ was applied to the column for 2 h at 4°C. Eight wash steps with TBS containing 10 mM Imidazole were performed to remove *E. coli* proteins which non-specifically interact. Captured proteins were then eluted with 250 μl of 1:1 TBS and Pierce Lysis Buffer containing 290 mM Imidazole. FlhF_His_ and L_2_ identification was performed by 10% SDS-PAGE followed by immunodetection using α-His and α-L_2_ (Beecher et al., 1995b) antibodies, respectively. To rule out the possibility that L_2_ had an intrinsic affinity for the cobalt resin or non-specifically interacted with *E. coli* proteins able to bind the resin, the soluble fraction of cells expressing L_2_ was directly applied to the equilibrated resin and to the resin previously incubated with the lysate of *E. coli* cells containing the empty vector pET303/CT. Both control samples were treated as described above.

### Flagella Staining and Flagellin Purification

WT and MP08 strains were grown to the late exponential growth phase in BHIG for 6 h at 37°C (OD_600_ of ∼2). For flagella observation, 10 μl of bacterial cells were directly stained with tannic acid and silver nitrate (Harshey and Matsuyama, 1994). Several samples were analyzed at 1,000 × magnification using an optical microscope (BH-2; Olympus, Tokyo, Japan). The extent of cell flagellation was analyzed as previously described (Calvio et al., 2005). Briefly, bacterial cultures were vigorously vortexed for 30 s, and harvested by centrifugation at 5,000 × *g* for 10 min at 4°C. Flagellar filaments were collected from supernatants by ultracentrifugation at 100,000 × *g* for 1 h at 4°C. Protein concentration was determined by Pierce^TM^ BCA Protein Assay Kit (Thermo Fisher Scientific), samples were standardized accordingly, and were subjected to 10% SDS-PAGE followed by Comassie Blue staining. Experiments were performed in triplicate in separate days and a representative result is shown.

### Preparation of *B. cereus* Supernatants

Protein samples were prepared by growing WT and MP08 cells to the late exponential growth phase in BHIG for 6 h at 37° C (OD_600_ of ∼2). Cells were normalized to the same OD_600_ and culture supernatants were collected by centrifugation at 10,000 × *g*, and added with Halt Protease Inhibitor Cocktail, EDTA-Free 1 × and 0.5 mM EDTA. Protein concentration was determined with Pierce^TM^ BCA Protein Assay Kit (Thermo Fisher Scientific). To exclude cell lysis, the activity of the cytosolic marker fructose-1,6-bisphosphate aldolase in culture supernatants was spectrophotometrically determined (Warth, 1980). Culture supernatants were concentrated using Microcon^®^ -10 centrifugal filter units (Merck KGaA). After being standardized for total proteins concentration, protein samples were subjected to 10% SDS-PAGE and electrotransferred on PVDF membranes for L_2_ immunodetection (Beecher et al., 1995b). All experiments were performed three times in separate days and a representative result is shown.

### Image Analysis

Densitometric analysis of Comassie blue stained gels and immunodetected filters were performed using a ChemiDoc^TM^ XRS+ System with a software version 6.0.1.34 (Bio-Rad, Berkeley, California). Relative quantification was performed using the WT bands as reference.

### *In silico* Analysis

Nucleotide and amino acid sequences in the FASTA format were retrieved from the European Nucleotide Archive^1^ and the UniProt databases (The UniProt Consortium, 2015), respectively. BLASTn^2^ was used for comparative analysis of nucleotide sequences obtained from sequencing. Compute pI/Mw tool (ExPASy Bioinformatics Resource Portal) was used for molecular weight prediction of amino acid sequences. The representation of *B. cereus* FlhF domains was realized using the Illustrator for Biological Sequences (IBS) tool (Liu et al., 2015).

### Statistical Analysis

Data were expressed as the mean ± standard deviation (S.D). Statistical analysis was done on GraphPad Prism version 8.0.2. For β-galactosidase quantification, the one-way analysis of variance (ANOVA) followed by the Tukey HSD test for multiple comparisons was applied. For qRT-PCR experiments, ANOVA followed by Dunnett’s multiple comparisons test was used by setting the WT values as control. For densitometric data, the two-tailored Student’s *t*-test for unpaired data was used. A two-sided *p*-value (*p*) < 0.05 was considered significant.

## Results

### Self-Interaction of *B. cereus* FlhF

**FIGURE 1:**
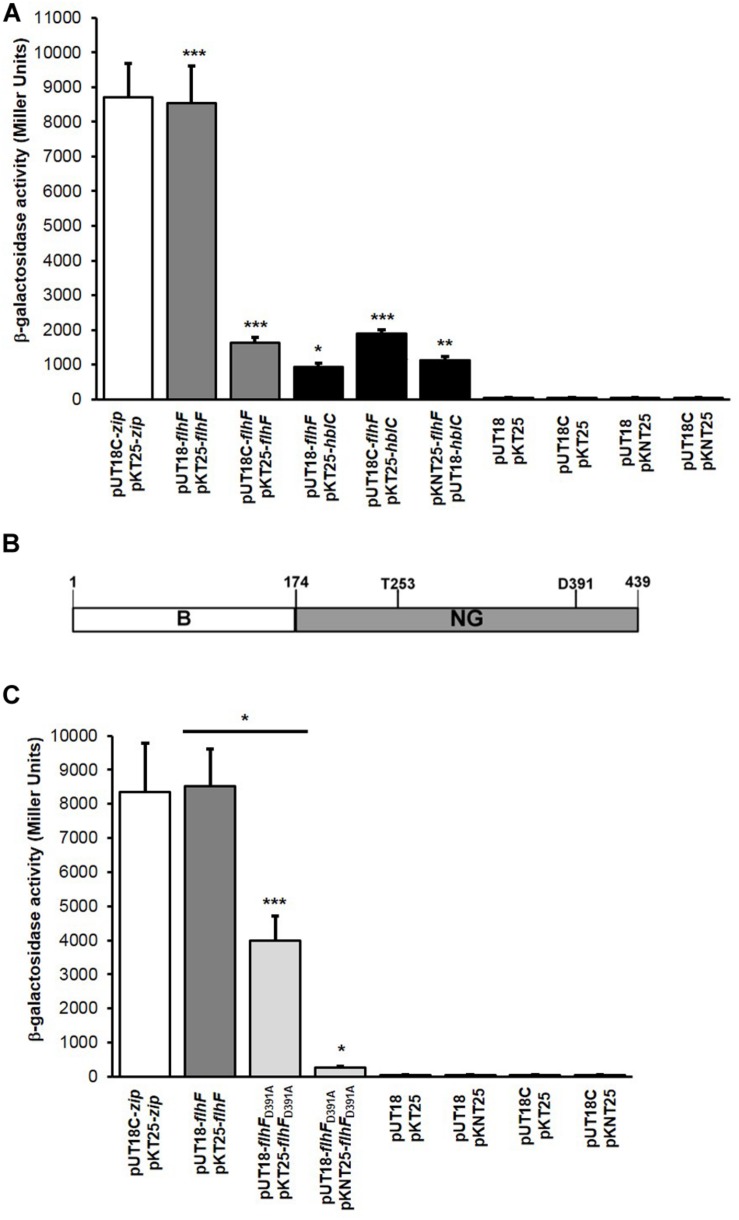
FlhF homodimerization, structure, and FlhF/L_2_ interaction. **(A)** Quantification of the β-galactosidase activity (reported in Miller Units) produced by positive clones. FlhF/FlhF interactions: gray bars; FlhF/L_2_ interactions: black bars; positive control: white bar; negative controls: dark gray bars. Values are expressed as the mean + S.D. from three independent experiments. ***p < 0.001, **p < 0.01, *p < 0.05, compared to the negative controls. **(B)** N-terminal B (white box, residues 1–173) and NG (light gray box, residues 174–439) domains of *B. cereus* FlhF. Threonine (T253) and aspartic acid (D391) residues potentially involved in protein homodimerization that were mutated by COE-PCR (T253Q and D391A) are shown. **(C)** Quantification of the β-galactosidase activity (reported in Miller Units) produced by *E. coli* clones grown on M63 medium. FlhF_D391A_/FlhF_D391A_ interactions: light gray bars; FlhF/FlhF interaction: gray bar; positive control: white bar; negative controls: dark gray bars. Values are expressed as the mean + S.D. from three independent experiments. ***p < 0.001; **p < 0.01, *p < 0.05.

The bacterial adenylate cyclase two-hybrid (BACTH) approach was applied to investigate whether FlhF forms dimers in *B. cereus*. The complete ORF of *B. cereus flhF* was fused to the T18 and T25 encoding domains of *Bordetella pertussis* adenylate cyclase (Cya) contained in pUT18, pUT18C, pKT25, and pKNT25 (Table 1), under the control of the *plac* promoter. Expression of recombinant pUT18 and pKNT25 vectors lead to the production of hybrid proteins in which the adenylate cyclase domain is localized in a C-terminal position. Fusion proteins expressed by recombinant pUT18C and pKT25 possess the enzymatic domain in an N-terminal location. Four combinations of recombinant plasmids (pUT18-*flhF*/pKT25-*flhF*, pUT18-*flhF*/pKNT25-*flhF*, pUT18C-*flhF*/pKT25-*flhF*, and pUT18C-*flhF*/pKNT25-*flhF*) were co-transformed into the reporter strain *E. coli* BTH101, which is defective for Cya activity. Fusion proteins were tested for their ability to interact by growing co-transformed cells on M63 minimal medium plates containing maltose as unique carbon source and IPTG as inducer of protein expression. Only in the case of FlhF/FlhF interaction, Cya could be restored, cyclic adenosine monophosphate (cAMP) synthesized, and the maltose operon genes as well as the cAMP-reporter gene β-galactosidase expressed in the *E. coli* BTH101 strain. Colonies containing the combinations of vectors pUT18-*flhF*/pKT25-*flhF* and pUT18C-*flhF*/pKT25-*flhF* were able to grow, indicating that a physical interaction between FlhF monomers exists *in vivo* when fusion proteins carried the T25 domain in a N-terminal position. These qualitative observations were supported by quantification of the β-galactosidase activity that was significantly increased (*p* < 0.001) compared to the negative controls (Figure 1A).

GTP-dependent homodimerization of *B. subtilis* FlhF has principally been attributed to a threonine (T184) and an aspartic acid residue (D320) found in the G1 and G4 elements of the G domain respectively, (Bange et al., 2007). To determine whether mutations in the corresponding residues of *B. cereus* FlhF (i.e. T253; D391; Figure 1B) altered self-dimerization, point mutations were generated by site-directed mutagenesis through combined overlap extension PCR (COE-PCR) to produce *flhF*_T253Q_ and *flhF*_D391A_ variants. BACTH experiments were separately performed with the two variants. No colonies were obtained on M63 plates with clones containing *flhF*_T253Q_, indicating that this residue is essential for FlhF dimerization (data not shown). In contrast, positive interactions were obtained with *E. coli* clones containing the combinations of vectors pUT18-*flhF*_D391A_/pKT25-*flhF*_D391A_ and pUT18-*flhF*_D391A_/pKNT25-*flhF*_D391A_ (data not shown). The β-galactosidase activity of these clones (Figure 1C) was higher than the negative controls (*p* < 0.001 for the combination pUT18-*flhF*_D391A_/pKT25-*flhF*_D391A_, and *p* < 0.05 for the combination pUT18-*flhF*_D391A_/pKNT25-*flhF*_D391A_), indicating that FlhF carrying the D391A substitution is still able to form dimers. However, β-galactosidase activity of *E. coli* clones containing pUT18-*flhF*_D391A_/pKT25-*flhF*_D391A_ was reduced compared to cells carrying the unmutated *flhF* in the same combination of vectors (*p* < 0.05; Figure 1C). This result suggests that, although dimers can be formed when FlhF carries the mutation D391A, interaction among mutated monomers is less stable than among monomers of wild-type FlhF.

### FlhF Is Required for Synthesis but Not for the Export of Flagellin

**FIGURE 2:**
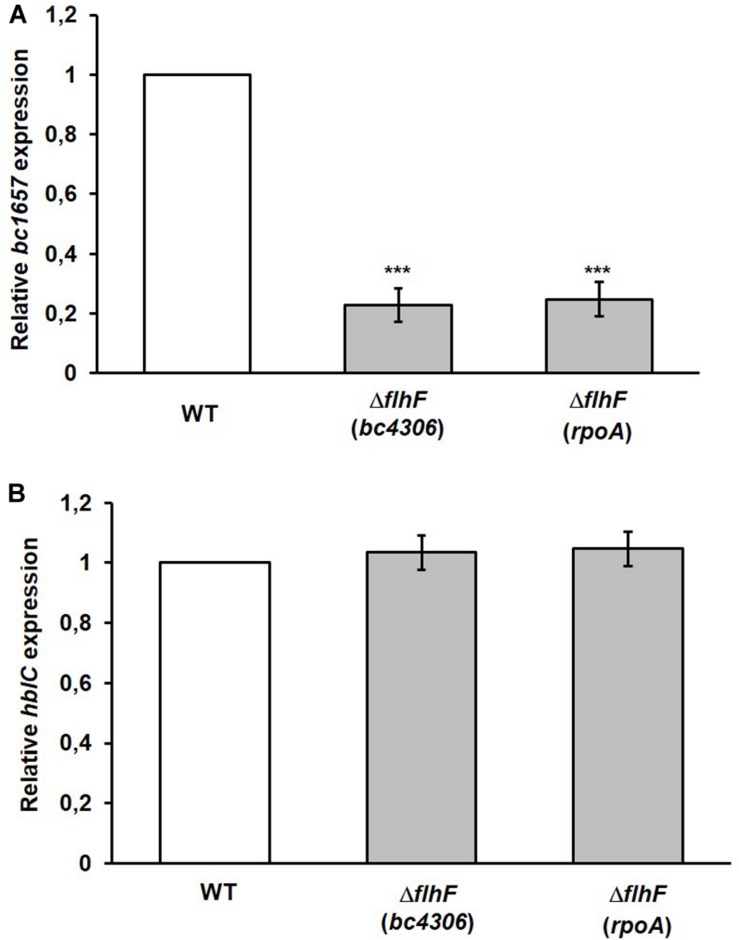
qRT-PCR analysis of the expression of flagellin (*bc1657*) and L_2_ (*hblC*) encoding genes in the Δ*flhF* mutant. **(A)** The level of *bc1657* expression in the Δ*flhF* mutant (light gray bars) is presented as fold change relative to the corresponding gene in the WT strain (*B. cereus* ATCC 14579; white bar). **(B)** The level of *hblC* expression in the Δ*flhF* mutant (light gray bars) is presented as fold change relative to the corresponding gene in the WT strain (*B. cereus* ATCC 14579; white bar). For both figures, *bc4306* and *rpoA* were independently used as reference genes for normalization. The relative *bc1657* and *hblC* expression in the WT (white bars) is conventionally set to 1. Bars represent means ± S.D. from three independent experiments. ***p < 0.001 compared to the WT.

Since the FlhF depletion in *B. cereus* causes a drastical reduction in the number of flagella (Salvetti et al., 2007), we performed a comparative analysis of the expression of the flagellin gene *bc1657* in the Δ*flhF* mutant and in the wild-type (WT) strain. As references, we used the genes *rpoA* and *bc4306*, encoding the DNA-directed RNA polymerase subunit alpha (Salvetti et al., 2011) and the Gatb_Yqey domain-containing protein (Reiter et al., 2011), respectively. For each strain, the threshold cycle (CT) of *bc1657* was separately normalized to the CTs of the reference genes. The transcription level of *bc1657* was lower in the Δ*flhF* mutant compared to the WT (*p* < 0.001) using both reference genes (Figure 2A). These findings indicate that FlhF is required for full *bc1657* expression in *B. cereus*.

To evaluate whether FlhF could directly be involved in flagellin targeting/recruitment to the plasma membrane, a physical interaction between FlhF and flagellin was checked *in vivo*. BACTH experiments were applied using eight combinations of recombinant vectors carrying the complete ORFs of *flhF* and *bc1657* (Table 1). No colonies were obtained on the M63 minimal medium indicating that FlhF is unable to interact with flagellin BC1657 *in vivo* (data not shown).

### FlhF Is Dispensable for the Expression of the L_2_ Encoding Gene

The low amount of the L_2_ component of HBL found in the extracellular proteome of the *B. cereus* Δ*flhF* strain (Mazzantini et al., 2016) prompted us to assess whether the transcription level of the L_2_ encoding gene (*hblC*) was altered in such a strain. A comparative expression analysis of *hblC* was performed in the WT and the Δ*flhF* mutant. Also in this case, *rpoA* and *bc4306* were used as reference genes. In contrast to flagellin, no significant difference (*p* > 0.05) in *hblC* expression was found using both reference genes, indicating that FlhF depletion does not alter *hblC* expression (Figure 2B). Therefore, the reduced amount of L_2_ secreted by the Δ*flhF* mutant (ratio Δ*flhF*/WT = 0.38) (Mazzantini et al., 2016) is not the result of an expression defect, but it likely seems the consequence of altered export.

### FlhF Physically Interacts With the L_2_ Component of HBL

To investigate on whether a physical interaction between FlhF and L_2_ occurs, BACTH experiments were performed using the eight combinations of recombinant vectors carrying the complete ORFs of *flhF* and *hblC* of *B. cereus* (Table 1). Positive interactions on the M63 minimal medium were obtained with clones containing the combinations of vectors pUT18-*flhF*/pKT25-*hblC*, pUT18C-*flhF*/pKT25-*hblC*, and pKNT25-*flhF*/pUT18C-*hblC*. The β-galactosidase activity of positive clones (Figure 1A) was significantly higher than the negative controls (*p* < 0.05 for the combination pUT18-*flhF*/pKT25-*hblC*; *p* < 0.001 for the combination pUT18C-*flhF*/pKT25-*hblC*, and *p* < 0.01 for the combination pKNT25-*flhF*/pUT18C-*hblC*). These findings indicate that FlhF directly interacts with L_2_ *in vivo*.

**FIGURE 3:**
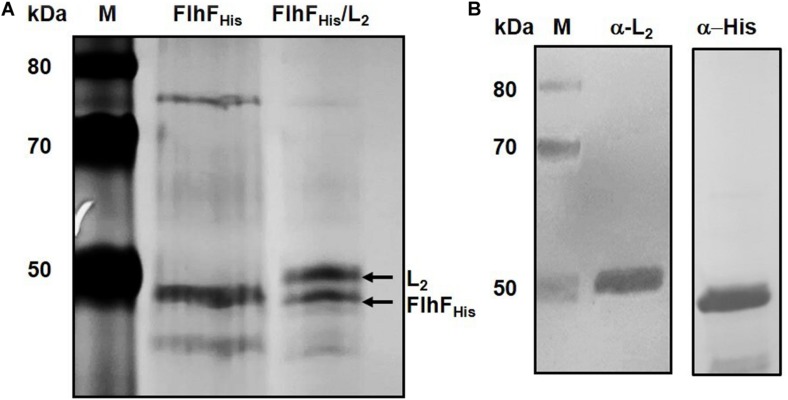
FlhF/L_2_ interaction tested by hexa-histidine pull-down. **(A)** SDS-PAGE of FlhF_His_ (lane 2) and FlhF_His_/L_2_ (lane 3) eluted fractions. **(B)** Immunoblot analysis of the FlhF_His_/L_2_ eluted fraction using anti-L_2_ (α-L_2_) and anti-His (α-His) antibodies. *M* = Thermo Scientific Spectra Multicolor Broad Range Protein Ladder (Thermo Fisher Scientific). Numbers on the left margins of the panels indicate the position of the molecular weight standards (kDa).

Hexa-histidine (His) pull-down was used as independent *in vitro* method to confirm FlhF/L_2_ interaction. A C-terminal His-tagged version of *B. cereus* FlhF (FlhF_His_) and an untagged version of L_2_ were expressed in *E. coli* BL21 (DE3) to form the bait and prey proteins, respectively. The soluble fraction of cells expressing FlhF_His_ was applied to the HisPur^TM^ Cobalt Resin and used as bait. The resin was washed and directly treated with elution buffer to obtain the FlhF_His_ fraction. In parallel, FlhF_His_-bound resin was incubated with the extract of cells expressing L_2_ and treated with the elution buffer to obtain the FlhF_His_/L_2_ fraction. Both fractions were subjected to SDS-PAGE. A band having a molecular weight compatible with FlhF_His_ was found in the FlhF_His_ and FlhF_His_/L_2_ fractions (Figure 3A). A second band with a molecular weight compatible with that predicted for L_2_ was observed only in the FlhF_His_/L_2_ eluted fraction. The identification of L_2_ and FlhF_His_ in the FlhF_His_/L_2_ fraction was performed by immunoblot analysis using anti-L_2_ (Beecher et al., 1995b) and anti-His antibodies, respectively. As shown in Figure 3B, immunoreactive bands corresponding to L_2_ and FlhF_His_ were found in this fraction. No FlhF_H__is_ and L_2_ immunoreactive bands were detected in controls samples (data not shown), indicating that L_2_ was pulled down only in the presence of FlhF_His_. Taken together, these findings indicate a direct interaction between FlhF and the L_2_ protein.

### The NG Domain of FlhF Interacts With L_2_

**FIGURE 4:**
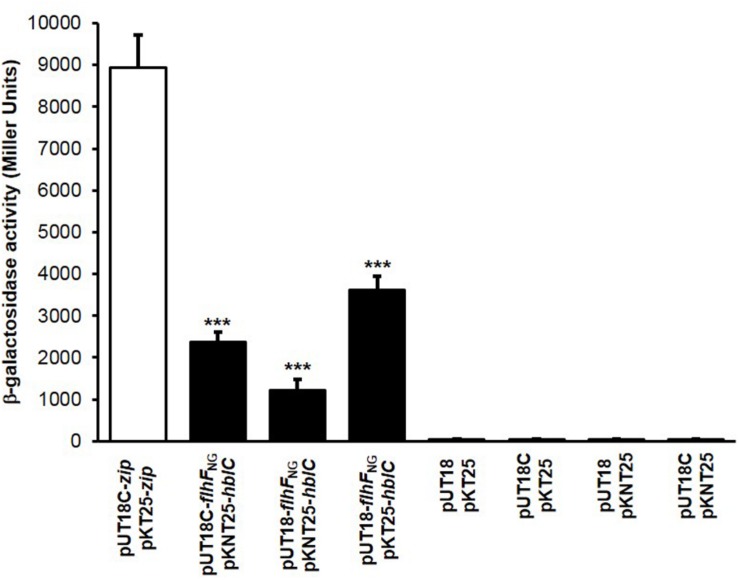
FlhF_NG_/L_2_ interactions tested by BACTH experiments. Quantification of the β-galactosidase activity (reported in Miller Units) produced by positive clones. FlhF_NG_/L_2_ interactions: black bars; positive control: white bar; negative controls: dark gray bars. Values are expressed as the mean + S.D. from three independent experiments. ***p < 0.001 compared to the negative controls.

To investigate if a physical contact between the NG domain and L_2_ can occur, the nucleotide sequence (*flhF*_NG_) encoding the NG domain of FlhF (Figure 1B) was amplified and cloned in the different vectors of the BACTH system (Table 1). The NG domain was tested for its ability to interact with L_2_ by co-transforming *E. coli* BTH101 with each of the above mentioned plasmids and each of the plasmids containing *hblC* (Table 1). The combinations of vectors pUT18-*flhF*_NG_/pKT25-*hblC*, pUT18-*flhF*_NG_/pKNT25-*hblC*, and pUT18C-*flhF*_NG_/pKNT25-*hblC* gave positive results on the M63 minimal medium. The β-galactosidase activity of these clones was significantly increased compared to the negative controls (*p* < 0.001; Figure 4). Based on these results, the NG domain of FlhF is able to interact with L_2_.

### Point Mutations in FlhF Impair L_2_ Binding

To identify a possible correlation between FlhF dimerization and L_2_ binding, BACTH experiments were performed. All the possible combinations of vectors containing *flhF*_T253Q_ and *hblC*, and *flhF*_D391A_ and *hblC* (Table 1) were used to transform *E. coli* BTH101.

None of the co-transformed *E. coli* cells grew on the M63 minimal medium (data not shown), indicating that no interaction of FlhF_T253Q_ and FlhF_D391A_ with L_2_ occurs and that both residues (T253 and D391) are essential for FlhF/L_2_ interaction. Taken together, our experiments suggest that the GTP-dependent homodimerization of FlhF is a prerequisite for its ability to interact with the L_2_ subunit of HBL.

### Effect of FlhF Overproduction on Flagella and L_2_ Secretion

**FIGURE 5:**
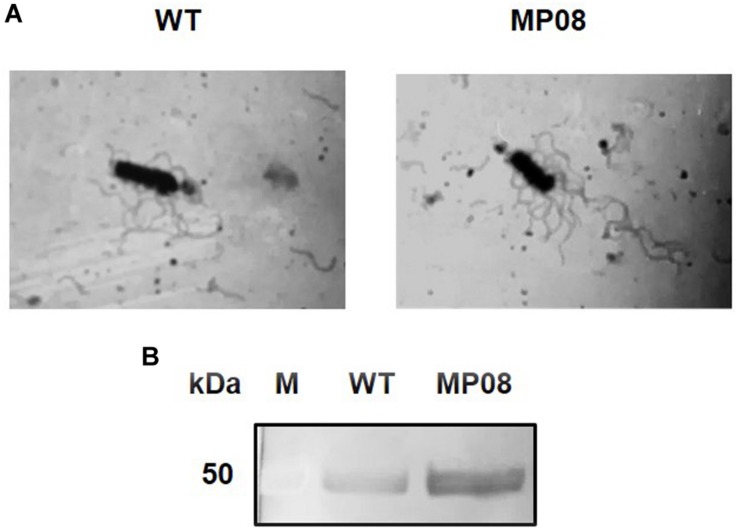
Effect of FlhF overexpression on *B. cereus* flagella and L_2_ secretion. **(A)** Light microscopy (1000×) of *B. cereus* ATCC 14579 (WT) and MP08 strain subjected to flagellar staining. **(B)** Immunodetection of the L_2_ component of HBL with rabbit polyclonal sera specific to L_2_. *M* = Thermo Scientific Spectra Multicolor Broad Range Protein Ladder (Thermo Fisher Scientific). Numbers on the left margins of the panels indicate the position of the molecular weight standards (kDa).

To evaluate the effect of FlhF overproduction on *B. cereus* flagellation and L_2_ secretion, a strain carrying a plasmid containing *flhF* under the control of the *pspac* promoter (MP08) was analyzed in comparison to the WT (Salvetti et al., 2007). Light microscopy of bacteria subjected to flagellar staining suggested an increase in the amount of flagella per cell in the MP08 strain compared to the WT (Figure 5A). Therefore, the extent of cell flagellation was analyzed by quantifying the amount of filament assembled flagellin in both strains. Density of the flagellin band was 1.83 ± 0.26-fold higher in the MP08 strain compared to the WT (*p* < 0.01; Supplementary Figure S1).

Normalized culture supernatants from the WT and MP08 were subjected to SDS-PAGE and immunoblot analysis for the detection of L_2_. The protein was more abundant (2.51 ± 0.171-fold higher) in the supernatant of the MP08 strain compared to the WT (*p* < 0.001; Figure 5B). Overall, these results indicate that FlhF overproduction increases the number of flagella and L_2_ secretion in *B. cereus*.

## Discussion

In the present study, we found that *B. cereus* FlhF is able to self-dimerize *in vivo*. Two of the four combinations of recombinant plasmids used to test FlhF dimerization indicated a positive interaction. The absence of interaction observed with the other combinations could be the consequence of a misfolding or instability of the hybrid proteins that prevent the contact, as previously suggested (Bange et al., 2011; Battesti and Bouveret, 2012).

FlhF homodimers are stabilized by hydrogen bonds *in trans* that involve both FlhF monomers (Bange et al., 2007). A threonine residue, conserved in all FlhF proteins with the exception of *P*. *aeruginosa* FlhF (Mazzantini et al., 2016), is believed to be essential for dimer stabilization through the interaction with the side chains of a lysine and a glutamic acid residue of the G4 element (Bange et al., 2007). However, the impact of the replacement of this residue on FlhF dimerization has never been analyzed before. Herein we demonstrate that a T253Q substitution in *B. cereus* FlhF completely abrogates homodimerization *in vivo*, indicating that this residue is essential for the interaction of monomers and/or for dimer stabilization.

In *B. subtilis* FlhF, specificity toward the guanosine nucleotides GDP and GTP is established by the interaction between an aspartic acid residue (D320 in *B. subtilis* FlhF) of the G4 element and the guanine base of the nucleotide with two hydrogen bonds. This residue is conserved in all SRP-GTPases (Bange et al., 2007). When a mutation was introduced in a residue corresponding to D320 in FtsY, the ability of the protein to bind and hydrolyze GTP was impaired, and FtsY could not form a complex with Ffh (Shan et al., 2004). A point mutation of the same aspartic acid residue strongly reduced the FlhF GTPase activity in *V. cholerae* and *S. oneidensis* (Green et al., 2009; Gao et al., 2015), and abrogated GTP binding and attenuated self-dimerization in *P. aeruginosa* (Schniederberend et al., 2013). The same mutation also impaired GTP load and dimer formation of FlhF in *V. alginolyticus* (Kusumoto et al., 2009; Kondo et al., 2018). In the present study, we found that the replacement of the corresponding D391 residue in *B. cereus* FlhF attenuates homodimerization, suggesting that this residue is required but not essential in this process. This result is in line with previous findings showing that a threonine residue of the G5 element (T343 in *B. subtilis*), conserved only in FlhF homologs, forms a third hydrogen bond with GTP in the active site, enhancing FlhF nucleotide binding compared to other SRP-GTPases (Bange et al., 2007). Since this residue is also conserved in *B. cereus* FlhF (T414) (Mazzantini et al., 2016), it could exert the same function maintaining a basal degree of GTP binding and FlhF dimerization.

Functionally, the main role of FlhF is supposedly related to flagellar assembly. The protein has been described to, directly or indirectly, drive the synthesis of flagellar components, stimulating wild-type levels of flagellar genes expression (Niehus et al., 2004; Kim et al., 2012; Kazmierczak and Hendrixson, 2013; Schuhmacher et al., 2015; Ren et al., 2018), and/or to dictate the point of flagellar assembly by recruiting early flagellar components to the plasma membrane (Murray and Kazmierczak, 2006; Green et al., 2009; Guttenplan et al., 2013; Altegoer et al., 2014). In the Δ*flhF* mutant of *B. cereus*, the amount of flagellin BC1657 was drastically reduced (ratio Δ*flhF*/WT = 0.22) (Mazzantini et al., 2016). In this study, qRT-PCR experiments were performed to analyze the transcriptional level of *bc1657* in the WT and the Δ*flhF* mutant using two housekeeping genes, *rpoA* and *bc4306*. *rpoA* has often been used as endogenous reference for qRT-PCR in *B. cereus* ATCC 14579 (Grande Burgos et al., 2009; Cadot et al., 2010; Salvetti et al., 2011). Since this gene has a high coefficient of variation (of about 43.0%), we also included the more stably expressed gene *bc4306*, which has a coefficient of variation of 8.41% (Bergman et al., 2006; Reiter et al., 2011). The finding that the transcription level of *bc1657* was significantly reduced in the Δ*flhF* mutant, suggests that FlhF is required for the regulation of flagellar gene expression in *B. cereus*. In line with this hypothesis, no physical interaction between FlhF and BC1657 was found *in vivo*. However, we cannot exclude that *B. cereus* FlhF could be involved in the recruitment of early flagellar basal body components to the plasma membrane, as previously indicated for other microorganisms (Green et al., 2009; Guttenplan et al., 2013) thus indirectly influencing the expression of late flagellar genes.

The striking similarities between FlhF and Ffh/FtsY might reflect similar functions in the targeting of proteins to the plasma membrane before secretion. Herein we demonstrate that the reduction in the export of the L_2_ component of HBL by the Δ*flhF* mutant (Mazzantini et al., 2016) is not due to a reduced expression of *hblC* and that physical interaction between FlhF and L_2_ occurs in *B. cereus*. FlhF interacts with the flagellar export chaperone FliS in *Helicobacter pylori* (Rain et al., 2001), with FlhG in *V. alginolyticus*, *B. subtilis* and *S. putrefaciens* (Kusumoto et al., 2008; Bange et al., 2011; Rossmann et al., 2015), with the pilus-twitching motility regulator XooPilL in *X. oryzae* pv *oryzae* (Shen et al., 2001), with the C ring protein FliG and the polar scaffolding protein FimV in *P. aeruginosa* (Schniederberend et al., 2019). In addition, the protein is required for the recruitment of the MS ring early flagellar component FliF in *V. cholerae* (Green et al., 2009). This is the first report showing evidence that FlhF interacts with a protein that is not involved in flagellar biogenesis or bacterial motility.

Little is known on the role of FlhF domains and on how these domains are involved in the binding of FlhF to its interactors. The G domain of FlhF was found essential in the recruitment of FliF to the cell pole in *V. cholerae* (Green et al., 2009), while the entire NG domain mediates the interaction between FlhF and FlhG in *B. subtilis* (Bange et al., 2011). In this study, we describe the L_2_ component of HBL as novel binding partner of FlhF in *B. cereus*. FlhF can cycle between two mutually exclusive forms: a cytoplasmic, monomeric, and nucleotide-free or GDP-bound, and a GTP-dependent, membrane-bound homodimer which represents the “ON” state of FlhF (Bange et al., 2007; Schuhmacher et al., 2015; Kondo et al., 2018). The dimeric form of FlhF has been described to be required for FlhF/FlhG contact and for the recruitment of flagellar components to the plasma membrane (Kusumoto et al., 2009; Bange et al., 2011; Schuhmacher et al., 2015; Kondo et al., 2018). Our finding that the mutant variants FlhF_T253Q_ and FlhF_D391A_ completely lose the ability to interact with L_2_ suggests that stable homodimerization of FlhF, which requires both T253 and D391, is crucial for L_2_ binding in *B. cereus*. Thus, we favor the idea that the GTP-bound homodimer of FlhF serves during secretion of the L_2_ subunit of HBL.

The effect of *flhF* overexpression on bacterial flagellation has been investigated in several microorganisms. Similar to polar flagellates (Pandza et al., 2000; Correa et al., 2005; Kusumoto et al., 2008; Green et al., 2009) and different to *B. subtilis* (Guttenplan et al., 2013), FlhF overproduction in *B. cereus* results in a hyperflagellated phenotype. FlhF levels are also critical in defining the amount of one extracellularly secreted HBL component, being therefore the protein important in modulating the pathogenic potential of *B. cereus*.

## Data Availability Statement

All datasets generated for this study are included in the article/Supplementary Material.

## Author Contributions

EG and GB designed the study. DMa and RF performed the experiments and prepared the figures. All authors analyzed the data and wrote the manuscript.

## Conflict of Interest

The authors declare that the research was conducted in the absence of any commercial or financial relationships that could be construed as a potential conflict of interest.

## Footnotes

1 https://www.ebi.ac.uk/ena

2 http://blast.ncbi.nlm.nih.gov/Blast.cgi

## Supplementary Material

FIGURE S1 Effect of FlhF overexpression on *B. cereus* flagellar filaments. SDS-PAGE analysis of extracellular flagellin of the WT and MP08 strains.

TABLE S1 Primers used for PCR amplification and/or sequencing.
