# Supplementary figures and images for: GTP-Dependent FlhF Homodimer Supports Secretion of a Hemolysin in Bacillus cereus
Source: Front Microbiol. 2020 May 6;11:879. doi: 10.3389/fmicb.2020.00879 (PMC7218170; doi:10.3389/fmicb.2020.00879)

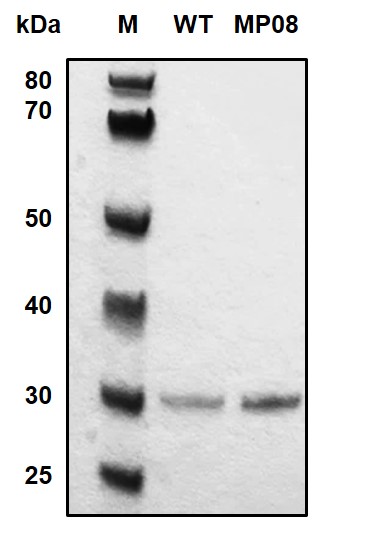

Supplement: FIGURE S1 — Effect of FlhF overexpression on B. cereus flagellar filaments. SDS-PAGE analysis of extracellular flagellin of the WT and MP08 strains. [file Image_1.JPEG]
