# Supplementary material for: GTP-Dependent FlhF Homodimer Supports Secretion of a Hemolysin in Bacillus cereus
Source: Front Microbiol. 2020 May 6;11:879. doi: 10.3389/fmicb.2020.00879 (PMC7218170; doi:10.3389/fmicb.2020.00879)
Supplement: TABLE S1 — Primers used for PCR amplification and/or sequencing. [file Table_1.DOCX]

**Supplementary Table S1: Primers used for PCR amplification and/or sequencing.**

| **Primer name^a, b, c^** | | **Sequence^d^** | **Restriction site** | **Reference** | |
| --- | --- | --- | --- | --- | --- |
| **For qRT-PCR analysis** | | | | | |
| *bc1657* Fup**^a^** | | CAAGCTACTAACATGGCTTC | None | This study |  |
| *bc1657* Rdw**^a^** | | TTTGGAAACCATTTGTGGAG |  | This study |  |
| hblL_2_ up**^a^** | | AAAGCAGCTCGTGAAGCAAT | None | Salvetti et al., 2011 | |
| hblL_2_ dw**^a^** | | GCAAAAACGCCAAATGTTTT | None | Salvetti et al., 2011 | |
| rpoAup1**^a^** | | GTATACGCCAGCTGATGCAA | None | Salvetti et al., 2011 | |
| rpoAdw1**^a^** | | ATGCTTCCATCCGTCCATAC | None | Salvetti et al., 2011 | |
| *gatB_Yqey* up**^a^** | | AGCTGGTCGTGAAGACCTTG | None | Reiter et al., 2011 | |
| *gatB_Yqey* dw**^a^** | | CGGCATAACAGCAGTCATCA | None | Reiter et al., 2011 | |
| **For BACTH experiments** | | | | | |
| *flhF*F1**^a^** | | ATAGGATCCGGGTGAAAGTAATG | BamHI | This study | |
| NGF1**^a^** | | GAAGGATCCTCAAGAATCTGTACCG | BamHI | This study | |
| *flhF* R1**^a^** | | CTTGGTACCGATGTTTGTAACAT | KpnI | This study | |
| *bc1657*F1^a^ | | GATGGATCCGGAGGTTCTTAACATGA | BamHI | This study | |
| *bc1657* R1^a^ | | ATGGGTACCTGTAATAATTTGGAAAC | KpnI | This study | |
| *L_2_* F2**^a^** | | CAAGGATCCAGGAGTGTACGGAATG | BamHI | This study | |
| *L_2_* R1**^a^** | | CAAGGTACCAATTTATATACTTGTTC | KpnI | This study | |
| *BcflhF*T253Q-F**^c^** | | GCATTAATCGGTCCACAAGGCGTTGGTAAAACG | None | This study | |
| *BcflhF*T253Q-R**^c^** | | CGTTTTACCAACGCCTTGTGGACCGATTAATGC | None | This study | |
| *BcflhF*D391A-F**^c^** | | GTGTTTACGAAATTTGCTGAAACAGCAAGTAGT | None | This study | |
| *BcflhF*D391A*-R***^c^** | | ACTACTTGCTGTTTCAGCAAATTTCGTAAACAC | None | This study | |
| *flhF*U3**^b^** | | ATGATTACGGAAGAAGAAGTT | None | Salvetti et al., 2007 | |
| *flhF*HindL4**^b^** | | TCAAAGCTTCCAATATGTACCTG | None | Salvetti et al., 2007 | |
| *bc1657*F2S**^b^** | | GTATTAAATTCTTAAGTACTGAT | None | This study | |
| *bc1657* R2S**^b^** | | TTGATATCAGCCGCACCACCCTT | None | This study | |
| L_2_F1S**^b^** | | TTACAGCAAATTGCAT | None | This study | |
| L_2_R1S**^b^** | | CTATTTTTCCTGTTCC | None | This study | |
| **For pull-down experiments** | | | |  | |
| PD*flhF*F1**^a^** | ATTTCTAGATAGGGGGTGAAGTAATG | | XbaI | This study | |
| PD*flhF*R2**^a^** | TAACTCGAGTTTTGCTAAATGTTCAGC | | XhoI | This study | |
| PDL_2_F1**^a^** | GGAATGCATGAATGAAAACTAAAATAATTA | | NsiI | This study | |
| PDL_2_R1**^a^** | AACCTCGAGTTAAAATTTATATACTTGTTCT | | XhoI | This study | |
| T7 Promoter**^c^** | TAATACGACTCACTATAGGG | | None | Thermo Fisher Scientific | |
| T7 Reverse**^c^** | TAGTTATTGCTCAGCGGTGG | | None | Thermo Fisher Scientific | |

^a^ These primers were used for both PCR amplification and sequencing.

**^b^** These primers were used only for sequencing.

**^c^** These primers were used only for PCR amplification.

^d^ Restriction site is underlined.
